# Risk of Hand Syndromes in Patients With Diabetes Mellitus: A Population-Based Cohort Study in Taiwan

**DOI:** 10.1097/MD.0000000000001575

**Published:** 2015-10-16

**Authors:** Lu-Hsuan Chen, Chung-Yi Li, Li-Chieh Kuo, Liang-Yi Wang, Ken N. Kuo, I-Ming Jou, Wen-Hsuan Hou

**Affiliations:** From the Department and Graduate Institute of Public Health (L-HC, C-YL, L-YW), College of Medicine, National Cheng Kung University, Tainan; Department of Public Health (C-YL), College of Public Health, China Medical University, Taichung; Department of Occupational Therapy (L-CK), College of Medicine, National Cheng Kung University, Tainan; Center of Evidence-Based Medicine (KNK, W-HH), Taipei Medical University, Taipei; Department of Orthopedics (I-MJ), National Cheng Kung University Hospital, Tainan; Master Program in Long-Term Care (W-HH), College of Nursing, Taipei Medical University, Taipei; School of Gerontology Health Management (W-HH), College of Nursing, Taipei Medical University, Taipei; and Department of Physical Medicine and Rehabilitation (W-HH), Taipei Medical University Hospital, Taipei, Taiwan.

## Abstract

The aim of this study was to assess the overall and cause-specific incidences of diabetic hand syndromes (DHS) in patients with diabetes mellitus (DM) by using age and sex stratifications.

The DM and control cohorts comprised 606,152 patients with DM and 609,970 age- and sex-matched subjects, respectively, who were followed up from 2000 to 2008. We estimated the incidence densities (IDs) of overall and cause-specific DHS, namely carpal tunnel syndrome (CTS), stenosing flexor tenosynovitis (SFT), limited joint mobility (LJM), and Dupuytren disease (DD), and calculated the hazard ratios (HRs) of DHS in relation to DM by using a Cox proportional hazards model with adjustment for potential confounders.

Over a 9-year period, 51,207 patients with DM (8.45%) and 39,153 matched controls (6.42%) sought ambulatory care visits for various DHS, with an ID of 117.7 and 80.7 per 10,000 person-years, respectively. The highest cause-specific ID was observed for CTS, followed by SFT, LJM, and DD, regardless of the diabetic status. After adjustment for potential confounders, patients with DM had a significantly high HR of overall DHS (1.51, 95% confidence interval [CI] = 1.48–1.53). Men and women aged <35 years had the highest HR (2.64, 95% CI = 2.15–3.24 and 2.99, 95% CI = 2.55–3.50, respectively). Cause-specific analyses revealed that DM was more strongly associated with SFT (HR = 1.90, 95% CI = 1.86–1.95) and DD (HR = 1.83, 95% CI = 1.39–2.39) than with CTS (HR = 1.31, 95% CI = 1.28–1.34) and LJM (HR = 1.24, 95% CI = 1.13–1.35).

Men and younger patients with DM have the highest risk of DHS. Certain hand syndromes, such as SFT and DD, were more strongly associated with DM than with other syndromes and require the attention of clinicians.

## Abstract 2

Supplemental Digital Content is available in the text

## INTRODUCTION

Diabetes mellitus (DM) is a chronic disease characterized by hyperglycemia, accompanied by several widely recognized complications, such as neuropathy, nephropathy, and retinopathy. In addition, certain musculoskeletal system complications, such as diabetic foot, occur and may result in immobility or amputation. However, musculoskeletal conditions of the upper extremities, particularly of the hands, called diabetic hand syndromes (DHS), have not gained adequate recognition.

DHS is a clinical condition occasionally occurring in patients with DM with a prolonged duration, suboptimal glycemic control, and peripheral vascular complications.^1–5^ Previous studies have proposed that DHS is characterized by several conditions, namely limited joint mobility (LJM) or diabetic cheiroarthropathy,^4^ trigger finger or flexor tenosynovitis (FTS),^3,6^ Dupuytern disease (DD),^6,7^ and carpal tunnel syndrome (CTS),^6,8,9^ and some of these conditions often coexist and can be potentially disabling. Nonetheless, the clinical pathoetiology for DM-related DHS incidence has not been fully elucidated.

Although some previous studies have reported DHS manifestations in patients with DM,^10,11^ information regarding the DHS incidence rate is scant. Moreover, whether the DHS incidence varies with the age and sex of patients with DM remains unknown. Therefore, we conducted a population-based cohort study for assessing the overall and cause-specific risks of DHS (ie, LJM, FTS, DD, and CTS) in patients with DM. In addition, we aimed to explore the age- and sex-specific relationships between DHS and DM.

## MATERIALS AND METHODS

### Data Source

This retrospective cohort study was performed using data from the Taiwan National Health Insurance Research Database (NHIRD). The Taiwan National Health Insurance (NHI) program, a mandatory, single-payer health insurance system for all residents of Taiwan, was implemented in 1995. According to the National Health Insurance Administration (NHIA) website (http://www.nhi.gov.tw), at the end of 2012, the NHI program covered 99% of the population of Taiwan (approximately 23 million people). In 2013, 93.7% of the clinics and hospitals in Taiwan, including >100 regional hospitals and tertiary referral medical centers, were contracted with the NHI program and provided medical services that were reimbursed by the NHIA. NHI medical claims data are routinely collected and supervised by the National Health Research Institutes (NHRI) to generate the NHIRD. Information that could be used to identify beneficiaries and medical care providers is scrambled by the NHIA. The NHIRD releases encrypted data to researchers for protecting patient and physician privacy. The NHIRD contains medical information regarding beneficiary characteristics, diagnosis and procedure codes for inpatient and outpatient care, medical orders, and medical expenditure. Because the NHIRD comprises deidentified secondary data released for research purposes, this study was exempted from full review by the Institutional Review Board. However, access to the NHIRD for our study was reviewed and received ethical approval from the NHRI Reviewing Committee (No. 93126), which ensures appropriate use of claims data. International Classification of Diseases, Ninth Revision, Clinical Modification (ICD-9-CM) codes were used to define diseases and procedures.

### Study Design and Subjects

This was a cohort study in which patients with DM and the control group were followed from 2000 to 2008. The study sample was chosen from a previous study, and sample selection details have been addressed previously.^12^ In brief, the DM cohort included all cases who received an outpatient DM diagnosis (ICD-9-CM: 250 or A-code: A181) at least twice between 2000 and 2001, and the control group comprised age- and sex-matched beneficiaries registered in 2000 who had never been diagnosed with DM from 1997 to 2000. We excluded patients who had medical claims for any hand syndromes from January 1, 1997 to the date of initial outpatient visit for DM treatment in 2000.

### Follow-Up and End Points

The index date for patients with DM was the date of initial diagnosis of DM in 2000. For the control cohort, it was July 1, 2000, or the first date of enrollment in the NHI program if the control subject enrolled after July 1, 2000. The follow-up period was initiated from the index date to the occurrence of the following hand syndromes: CTS (ICD-9-CM: 354.0), stenosing flexor tenosynovitis (SFT; ICD-9-CM: 727.03), LJM (ICD-9-CM: 718.8), and DD (ICD-9-CM: 728.6) in patients with DM. For those who did not experience any hand syndromes, the time of censoring was the date of insurance termination or the end of follow-up (December 31, 2008).

## COVARIATES

In addition to sex and age, we considered the geographic area and urbanization level as covariates in the analysis. Adjustment was performed for geographic area and urbanization level to minimize the potential confounding by differential accessibility and availability of medical care.^13^ Adjustment for occupation and insurance premium (an indicator of paid salary) was performed for considering the potential influence of work-related hazards on hand syndromes. Additional covariates included certain clinical risk factors for hand syndromes, such as epilepsy (ICD-9-CM: 345 and 780.39), arthropathy and rheumatoid arthritis (ICD-9-CM: 711, 714, 716, 719, and 729), and overweight and obesity (ICD-9-CM: 278).^14–16^ Information regarding clinical conditions was retrieved from the medical claims between January 1, 1997 and the index date.

### Statistical Analysis

The characteristics of the 2 study groups were described using counts and percentages and compared using the χ^2^ test. Incidence densities (IDs) of all-cause and cause-specific hand syndromes were calculated by dividing the number of people who sought medical care for hand syndromes by the total of person-years observed. The confidence interval (CI) of ID was estimated using Poisson distribution. Proportional hazard regression models were used for estimating the hazard ratio (HR) of all-cause and cause-specific hand syndromes between the 2 study groups. In addition to estimating the HR for the entire study sample, we performed sex- and age-stratified analysis to examine the potential effect-modifications by sex and age on the association between DM and the risk of hand syndromes. Data management and analyses were performed using SAS (Version 9.3, SAS Institute Inc, Cary, NC). A *P* value <0.05 was considered statistically significant.

## RESULTS

Sex, age, geographic area, and urbanization levels were similar in both groups. The prevalence rates of the risk factors for hand syndromes were high in patients with DM. The DM cohort had fewer white-collar workers (15.98% vs 18.09%), a higher prevalence of dependence (37.48% vs 34.05%), a lower insurance premium, and a higher frequency of ambulatory care visits (34.17 vs 20.76 per year) (Table 1) than the control cohort.

**TABLE 1:**
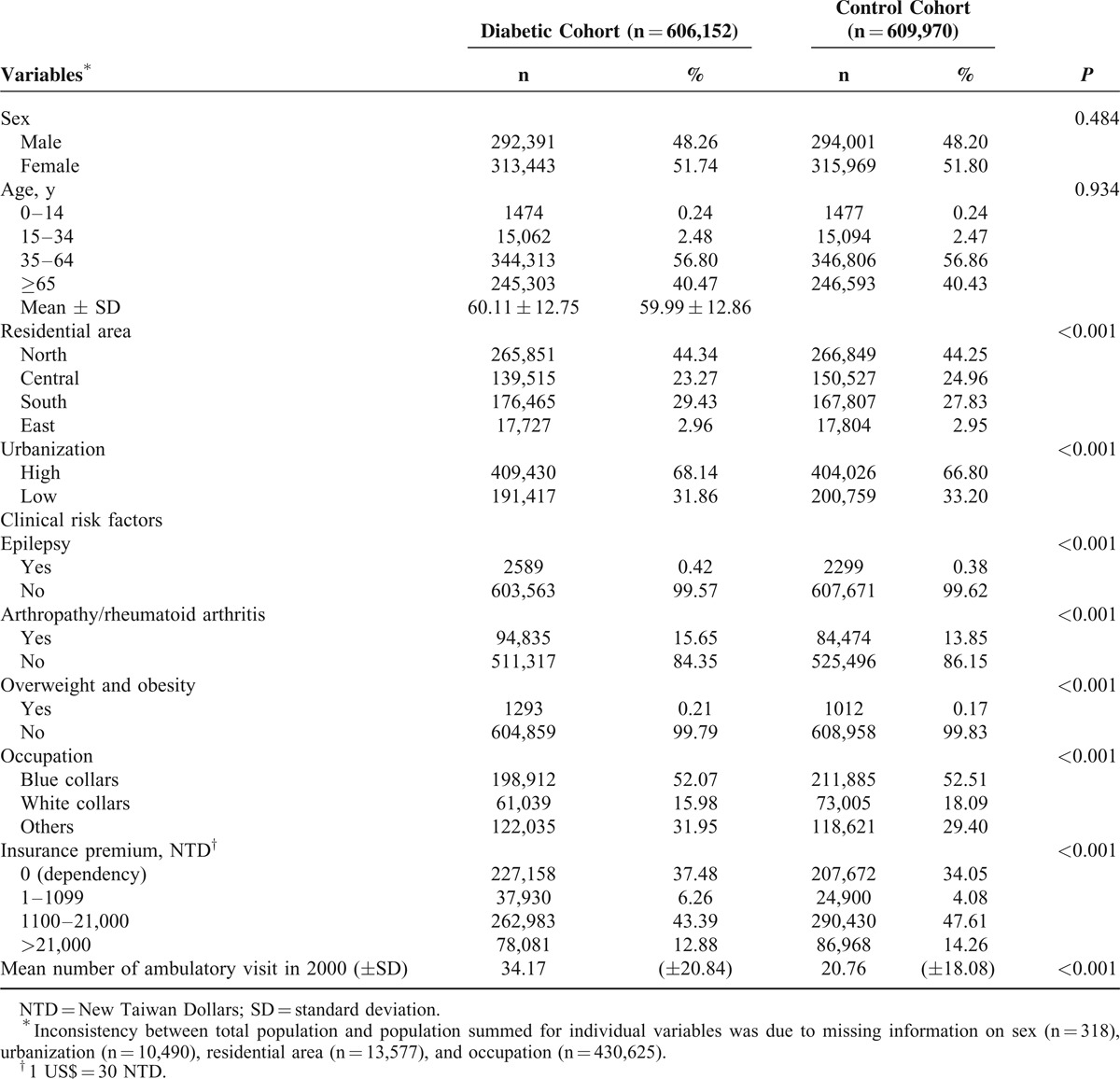
Characteristics of Study Cohorts

Over the 9-year follow-up period, 51,207 patients with DM and 39,153 controls developed various hand syndromes, with an ID of 117.7 (95% CI = 116.7–118.8) per 10,000 person-years. The overall covariate-adjusted HR of hand syndromes in relation to DM was 1.51 (95% CI = 1.48–1.53), with a significantly higher adjusted HR in men than in women (1.57 [95% CI = 1.52–1.61] vs 1.48 [95% CI = 1.44–1.51], *P* = 0.0008). The interaction of DM with age was statistically significant for both men and women (*P* < 0.0001 for both sexes), indicating that younger patients had a higher adjusted HR (Table 2).

**TABLE 2:**
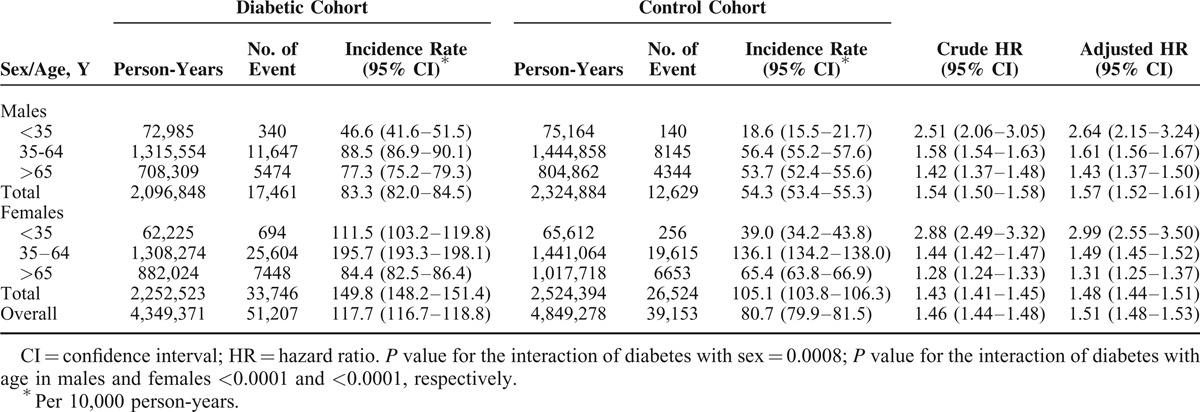
Overall and Sex- and Age-Specific Incidence Rate and Hazard Ratio of Having Hand Syndrome(s) Associated With Diabetes

Cause-specific analyses revealed that the IDs of CTS, SFT, LJM, and DD for patients with DM were 63.5 (95% CI = 62.8–64.3), 57.5 (95% CI = 56.8–58.2), 3.7 (95% CI = 3.5–3.9), and 0.40 (95% CI = 0.34–0.46) per 10,000 persons, respectively, and the values were significantly higher than those of the controls, with the adjusted HR values of 1.31 (95% CI = 1.28–1.34), 1.51 (95% CI = 1.48–1.53), 1.24 (95% CI = 1.13–1.35), and 1.83 (95% CI = 1.39–2.39), respectively. DM was significantly associated with sex only for CTS, and DM-related CTS was higher in men than in women (1.38 [95% CI = 1.33–1.43] vs 1.27 [95% CI = 1.24–1.31]). Moreover, effect-modification by age was significant for CTS and SFT, in which younger people had a higher adjusted HR irrespective of the sex (Supplementary Tables 1–4, http://links.lww.com/MD/A438).

## DISCUSSION AND CONCLUSIONS

This population-based cohort study revealed that patients with diabetes had a significantly higher risk of DHS compared with the control group, particularly in men and people aged <35 years. For cause-specific HR, both SFT (1.90) and DD (1.83) were the leading causes of diabetes-related DHS. To the best of our knowledge, only one previous study has estimated the incidence of trigger finger after CTS release in patients with DM.^17^ Our study is the first to report the incidence rates of overall and cause-specific DHS according to various age and sex stratifications.

Several mechanisms have been proposed to explain the association between DM and DHS. Patients with DM may experience certain musculoskeletal complications because of macrovascular and microvascular complications.^18,19^ An increase of advanced glycosylation end-products (AGEs) may result in skin thickening and formation of nodules and contractures.^20,21^ In addition, unregulated proliferation of collagen may also result from irregular expression of peptides that regulated a number of growth factors including cytokines transforming growth factor-β^22^ and basic fibroblast growth factor.^21^ Moreover, abnormal fibroblast proliferation and matrix production as well as an increase in matrix proteoglycans and free radicals in the affected tissues may all contribute to an increased risk of DHS in DM.^21,23,24^ Accordingly, damaged vessels or nerves, protein glycosylation, and increased collagen deposition in the skin and musculoskeletal connective tissues are factors possibly contributing to DHS complications in DM.^19,25^ Several previous studies have reported the positive association between the occurrence of DHS and the disease duration of DM, retinopathy, neuropathy, or nephropathy.^10,26,27^ Therefore, the relationships between hand soft tissue lesions and DM can be caused by the following 2 mechanisms: a primary soft tissue lesion directly affected by DM and secondary soft tissue complications caused by diabetic vasculopathy or neuropathy.

The IDs of DHC subclassifications differed in the DM and control groups, in which CTS had the highest ID, followed by SFT, LJM, and DD. A high CTS ID may be caused by the glycation of the connective tissue and diabetic neuropathy, thus contributing to CTS together or individually.^27^ Although the accumulation of AGE in collagen has been proposed as the common underlying cause of LJM, FTS, and DD, the variety of soft tissue locations with damaged collagen may lead to different incidences of DHS subclassifications. From the broader to the narrower areas of the soft tissue lesion, LJM is characterized by thickened and tightened skin and tendon sheaths, causing inability to fully flex or extend fingers.^2^ FTS causes tendon sheath swelling, forcing fingers into a flexed position.^28^ DD is an inherited proliferative connective tissue disorder involving the palmar fascia.^29^

Furthermore, men aged <35 years had a higher adjusted HR for DHS. Knowledge of the HR for different age and sex groups is vital in daily diabetic practice. Alarmingly high DHS HR end points in patients with DM aged <35 years may contribute to emerging health problems in Taiwan. A previous study proposed that younger patients with DM had poor glycemic control, poor health-related behaviors, fewer clinic visits, and less regular assessment of diabetes-related complications.^30^ This could lead to high HRs of various DHS among younger DM patients. Another study supported the underlying explanation that men had lower awareness of self-health to seek medical attention than women, thus leading to underestimation of DHS incidence at the baseline.^31^ Accordingly, a more aggressive approach is essential for screening and treating younger men with DM, who are vulnerable to DHS complications.

This study had the following strengths. First, it was a population-based study of a highly representative sample of patients with diabetes in Taiwan in the year 2000. Second, the advantage of using insurance claims data in clinical research is the easy access to longitudinal records for a wide sample of demographically diverse patients.^32,33^ The size of the data set enabled stratified analyses of certain demographic variables of interest, such as age and sex. Third, this DM cohort was formed using the NHI database, and all the research information was retrieved from NHI claims, which has a low rate of nonresponse or loss to follow-up.

Our study had several limitations. First, exclusive reliance on claims data may have resulted in a potential disease misclassification bias. The DHS incidence estimated from the claims data could be biased because some people who experience DHS-related symptoms may not seek ambulatory care, which would in turn lead to underestimation. Similarly, some people may be incorrectly diagnosed with DHS because of their increased interaction with the health care system for their hand condition (ie, surveillance bias). To address this concern, we calculated the number of ambulatory visits and adjusted for it in the multivariate regression model. Second, we were unable to consider a comprehensive list of potential confounders in the analysis, which may have resulted in residual confounding in our study. The unadjusted potential confounders may include certain socioeconomic factors (including occupation and financial status)^34^ and health behaviors (such as glucose control and smoking),^29,35^ which have been proposed to cause variations in the risk of musculoskeletal-related hand syndromes. In addition, DM duration is a strong potential confounder for the risk of DHS^19^; however, information regarding DM duration is unavailable in the NHI claims data. Third, we did not individually analyze the incidence of DHS according to patients diagnosed with type 1 or type 2 DM mainly because both ICD-9-CM and A-codes were used in the NHI disease coding system at the outpatient settings. Differentiating between type 1 and type 2 DM by using A-codes alone is not possible. Because only 1.8% of DM patients in Taiwan have type 1 DM,^36^ the majority of patients in our study were likely to have type 2 DM.

Using the 1997 diabetes prevalence rate, we calculated the population attributable risk percentage (PAR%) to assess the public health impact of diabetes on overall and cause-specific DHS. The PAR% for DHS, CTS, SFT, LJM, and DD were 2.88%, 2.98%, 6.22%, 0.49%, and 4.45%, respectively. In conclusion, this study demonstrated that men and younger patients with DM had the highest risk of DHS. Although the risk of overall DHS only modestly increased in patients with DM, certain hand syndromes, such as SFT and DD, were strongly associated with DM. Therefore, these findings highlight not only the objects that require the attention of clinicians, but also the necessity for subsequent genetic studies regarding DHS.
